# Volume reduction of benign thyroid nodules 3 months after a single treatment with high-intensity focused ultrasound (HIFU)

**DOI:** 10.1186/s40349-015-0024-9

**Published:** 2015-03-04

**Authors:** Huedayi Korkusuz, Niklas Fehre, Michael Sennert, Christian Happel, Frank Grünwald

**Affiliations:** Department of Nuclear Medicine, University Hospital Frankfurt, Theodor-Stern-Kai 7, 60590 Frankfurt am Main, Germany; German Center for Thermoablation of Thyroid Nodules (Deutsches Zentrum für Thermoablation von Schilddrüsenknoten), 60590 Frankfurt am Main, Germany

**Keywords:** High-intensity focused ultrasound ablation, Thyroid nodule, Ablation techniques, Volume reduction

## Abstract

**Background:**

High-intensity focused ultrasound (HIFU) is a promising, non-invasive technique in treating benign thyroid nodules (TNs). The aim of this study was to evaluate the efficacy of HIFU to induce clinically meaningful shrinkage in benign predominantly solid TNs and to identify variables that influence or predict the magnitude of TN volume reduction.

**Methods:**

For each of ten subjects, HIFU treatment was conducted on a single nodule. Nodular volume was measured sonographically at baseline and at 3 months post-procedure. Nodular function and early treatment assessment was done scintigraphically.

**Results:**

Median nodular volume reduction was 0.7 ml absolute and 48.8% relative to pre-interventional size (*p* < 0.05). Absolute shrinkage was negatively correlated with the average treatment depth (*τ* = −0.61, *p* < 0.05). Absolute nodular volume was positively correlated with the scintigraphic nodular uptake reduction (*τ* = 0.66, *p* < 0.05).

**Conclusions:**

HIFU treatment of benign predominantly solid TNs appears to be safe and effective for inducing nodular shrinkage. Despite potential for improvement, a single treatment session with HIFU is already a viable alternative to more standard methods. The feasibility of multiple HIFU treatments requires further investigation. Due to the small sample size, the findings of this analysis need conformation by larger studies.

## Background

Thyroid nodules (TNs) are common in western populations, most being of a benign nature [1]. Apart from autonomous nodular overproduction, all issues related to these nodules, such as swallowing disorders, throat tightness, hoarseness, neck pain or discomfort, are directly caused by TNs’ volume which puts pressure on neighbouring anatomical structures. Additionally, cosmetic problems associated with TNs are directly related to the nodules’ volume.

For many years, surgery and radioiodine therapy (RIT) were the only options for treatment of benign TNs. Both procedures are costly and the former is not without risk [2]. Moreover, some patients are uncomfortable with these methods due to their invasive or radioactive nature [3].

Additional techniques that have been widely discussed in recent years include ethanol injection into the TN (ethanol ablation (EA)) [4,5], thermal destruction of the nodular tissue via the application of microwave ablation (MWA) [6,7], interstitial laser photocoagulation (ILP) [8-10] and radio-frequency ablation (RFA) [11,12]. Several studies report that these methods are effective and have low complication rates [13]. Such techniques are minimally or not at all invasive and, in many cases, are less costly than surgery or RIT. However, relative to thyroidectomy or RIT, assessments are lacking of these methods’ risks, costs, long-term clinical outcomes and patient satisfaction [9].

To date, use of non-invasive tissue ablation by high-intensity focused ultrasound (HIFU) has been reported in a number of indications including uterine fibroids [14], prostate cancer [15] and benign breast lesions [16], and is under development for liver and kidney tumours [17].

To our knowledge, the application of HIFU to TN has only been reported in two preclinical studies [18,19] and one human case study [20]. The method’s ability to induce locally limited ablation, as opposed to efficacy, was evaluated histologically in a study where patients were treated with HIFU prior to scheduled thyroidectomy [21].

In living human tissue, temperatures between 60°C and 100°C induce coagulation necrosis without vaporization and carbonation [22]. Thermal ablation uses this method without inflicting damage to surrounding structures, providing a method to inflict precise localized destruction of the target structure. In the case of HIFU, this can be done even without surgical penetration of the skin. As ablated tissue is disintegrated over time by immunology cells, the intervention can cause a significant shrinkage of the target structure.

The objectives of this study were to quantify 3-month shrinkage of benign TNs in humans resulting from a single HIFU treatment session and to characterize TN features having potential to promote and/or predict clinically meaningful reduction in nodule volume.

## Methods

### Recruitment

This study was limited to ten patients only. According to inclusion and exclusion criteria, the first ten recruits could all be included in the study. Recruitment was closed afterwards. Recruitment took place in late 2013, and all treatment was done in January 2014. As one patient received therapy additional to HIFU treatment, this analysis is based on data of the nine remaining patients.

### Inclusion criteria

Patients over 18 years of age with at least one benign thyroid nodule and associated issues such as neck pain, hoarseness, swallowing disorders, discomfort, cosmetic concerns and/or thyrotoxicosis, who either refused surgery or RIT or who were contraindicated for them, were included in the study.

### Exclusion criteria

Patients with malignant nodules were excluded. Malignancy was diagnosed by atypical findings in preliminary ^99m^Tc-MIBI scintigraphy and confirmed by histological indication of follicular proliferation based on fine-needle aspiration biopsy (FNAB). Also excluded were patients with target nodules close to sensible structures like the trachea, oesophagus, recurrent nerve and carotid artery. Patients who showed any contraindication to HIFU were excluded. Contraindications are recurrent nerve anomalies and target volumes not clearly circumscribable in B-mode ultrasound (US).

If a patient presented with multiple TNs, the one chosen for treatment was the one primarily responsible for patient symptoms.

HIFU treatment was presented as an alternative to MWA, and patients were informed of known procedure risks (pain, recurrence of the treated nodule, vocal cord palsy, skin burn, tracheal and oesophageal injuries). All participating patients gave written informed consent. The study was approved by the ethics commission of the University Hospital Frankfurt.

### Equipment

HIFU treatment was conducted with the US-guided EchoPulse® (Theraclion SA, Malakoff, France). The guiding US operates at 7.5 to 12 MHz, and the therapeutic HIFU emits pulses at 3 MHz with a maximum sound power of 125 W. Both guiding and therapeutic US are emitted by a probe held by a robotic arm. The probe emitting the imaging US is placed in the middle of the HIFU emitter, so that the focal point of the therapeutic beam is always displayed in the centre of the guiding US image.

The physician plans treatment by defining the target area and sensible structures at about 10 to 20 sagittal and transversal section plane images of the guiding US at the EchoPulse® user interface. Security margins of 2 mm to the carotid, 5 mm to the skin and at least 3 mm to the trachea are automatically created by the EchoPulse®, and treatment in those zones is blocked. After treatment and non-treatment areas are defined, the HIFU device proposes a layer of treatment voxels. These so-called treatment sites have the approximate form of a rice corn; their long sides adjoin each other. Treatment sites are displayed within the guiding US image and can be deselected manually and thus excluded from treatment at any time.

Each HIFU pulse of 4-s duration is directed at one site within the nodular target volume, a focusing that is capable of inducing temperatures between 60°C and 80°C at the target ellipsoid site (diameter 2 mm, length 9 mm). As treatment sites directly border one another, the distance between two focal point centres is 2 mm. During the inter-pulse cooling period, the robotic arm redirects the probe at the next site. Cooling is provided by a circulating liquid of 10°C within a plastic balloon positioned at the forefront of the probe. This cooling of the skin above the treatment area prevents pre-focal tissue damage.

To avoid incongruency between planning and actual treatment, a laser-based distance measurement is implemented. This system automatically stops treatment if changes above 1 mm in the span between the robotic arm holding the probe and laser and the patient’s skin are registered.

### Procedure

The treatment was planned and carried out with the EchoPulse® user interface by a trained physician. To ensure sufficient heating in the target area without applying more potentially harmful energy than necessary, initial test pulses were emitted to adjust US intensity to just below the point where the so-called US-detectable “heat bubbles” indicate sufficient heating of TN tissue to induce necrosis.

If tissue is sufficiently heated, decentralized development of steam leads to the formation of the so-called heat- or microbubbles. Wood et al. [23] report that US-detectable microbubble generation in different human types of tissue starts somewhere between 80°C and 90°C.

**Figure 1:**
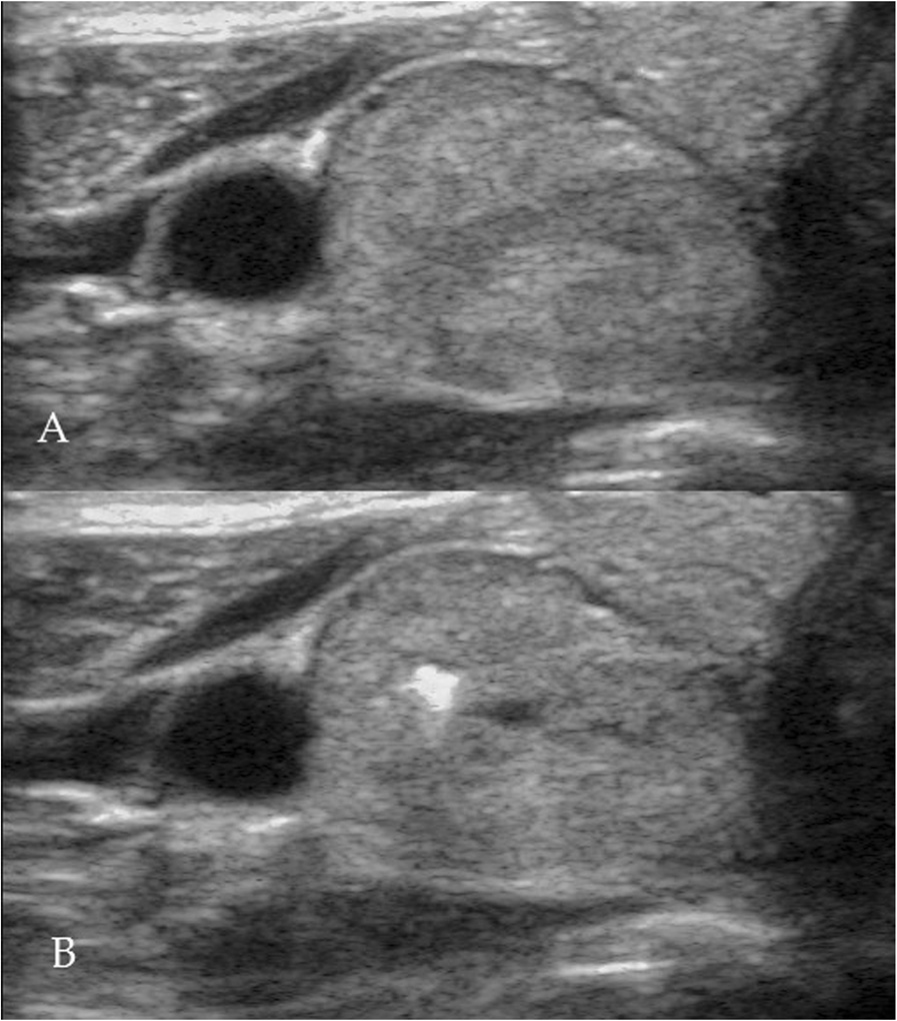
**Visual feedback during initial test pulse.** During initial test pulses, beam intensity was set to be just below the point where the generation of a so-called hyperechoic mark (HEM) indicates temperatures above 80°C–90°C. This mark is visible in B-mode US due to the hyperechogenic character of microbubbles. These consist of steam. Shown is an exemplary nodule before **(A)** and directly after **(B)** such a test pulse. Clearly visible is the HEM, which vanishes within seconds afterwards.

The detection of heat bubbles thus indicates temperatures of around 80°C–90°C, while only temperatures of above 60°C are necessary to induce the desired necrosis. To limit the applied energy (which always needs to pass through overlying tissue that we wanted to stay unharmed), we operated the HIFU just below the point of heat bubble formation. Figure 1 shows an exemplary nodule before and immediately after an initial test pulse. The clearly visible so-called hyperechoic mark (HEM) consists of microbubbles and indicates temperatures above 80°C.

During treatment, the planned outlines of the target area and sensible structures are displayed at all times, as are the actual anatomical localizations due to the underlying B-mode guiding US imaging. A potential deviation of the planned treatment grid and reality, if not detected by the laser measurement, can be easily spotted by the performing physician. In this case, the treatment can be manually interrupted until congruency is restored by probe repositioning. Such probe readjustments may be done manually or via steering the robotic arm using the interface. Inter-pulse probe repositioning to target the next treatment site is done automatically.

### Baseline evaluation and endpoints

Before HIFU treatment, nodules were assessed for size, structure and exact location using B-mode US imaging. Functional activity was evaluated using ^99m^Tc-pertechnetate scintigraphy (75 MBq, imaging 20 min post-administration with a thyroid-specific scintillation camera (Mediso Nucline® TH/22, acquisition time: 300 s, matrix: 128 × 128 × 16, low-energy collimator)). Nodules presenting as hypofunctional in this imaging were further examined using ^99m^Tc-MIBI (441 MBq, imaging 10 and 60 min post-administration with the same camera mentioned above, with 500-s acquisition time and 128 × 128 × 16 matrix) as well as FNAB cytology. Malignancy could be ruled out in all cases.

**Figure 2:**
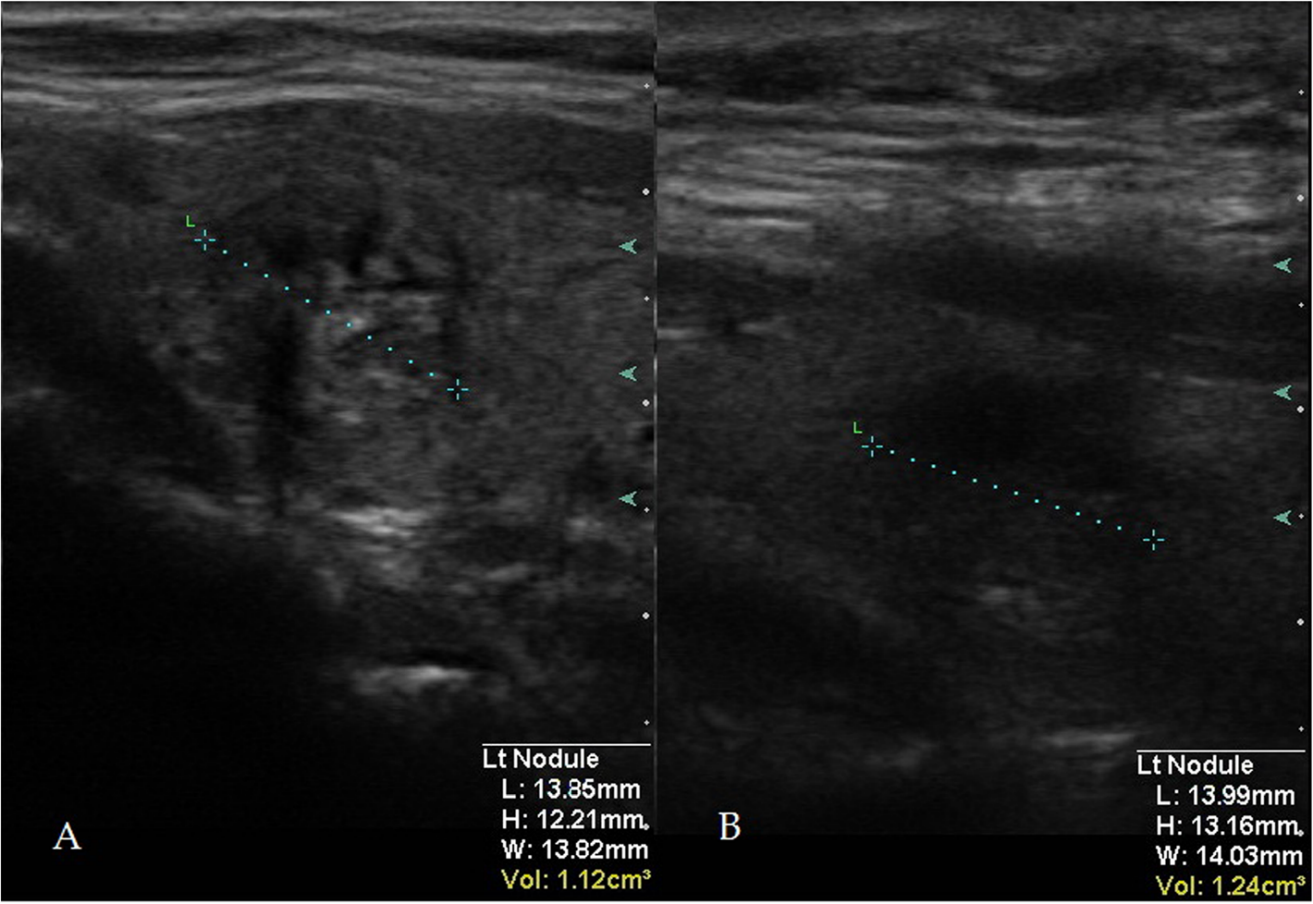
**Short-term change of nodule appearance due to HIFU treatment.** An exemplary nodule in B-mode US before **(A)** and directly after the completion of HIFU treatment **(B)**. An estimation of ablation magnitude is not possible due to reactive edema formation.

Due to reactive edema formation, ablative effects of the HIFU therapy are not quantifiable sonographically immediately after intervention (see Figure 2). Early assessment of treatment was done by scintigraphic means 1 day after the procedure. Scintigraphic imaging was done according to guidelines.

Nodular volume was measured again at 3 months post-ablation by a sonograph using the formula *V* = *h* × *w* × *d* / 2.09, with *h*, *w* and *d* representing height, width and depth, respectively. All sonographic examinations were done by KH and performed with a Sonix Touch Ultrasound system (Ultrasonix Medical Corporation, Richmond, Canada). Maximal nodular dimensions were measured in transversal and sagittal section plane B-mode US images. QA was ensured as all measurements were validated by a radiologist with specific sonographical experience.

The effect of HIFU treatment on nodule volume in terms of difference between 3 months as baseline was quantified in both absolute and relative terms (i.e. percentage of baseline).

Scintigraphic uptake reduction is reported as the relative target area background adjusted uptake reduction in relation to the total thyroidal uptake. These are measured within the region of interest using ^99m^Tc-pertechnetate for hot and indifferent nodules and ^99m^Tc-MIBI for cold nodules.

Procedure characteristics recorded by EchoPulse® and provided in treatment reports included treatment depth, procedure time, total energy delivered per patient and energy delivered to the target site.

### Statistics

Statistical analysis was done with the free R statistical software (R Core Team 2013); http://www.R-project.org/). Due to the small sample size, the test for a significant decrease of nodular volume was performed with non-parametric methods (one-sided Wilcoxon signed rank test). Significance is defined as *p* < 0.05.

Due to the small sample size and unknown underlying distributions, correlations are reported using Kendall’s *τ* and tested against the null hypothesis of *τ* = 0. Significance is defined as *p* < 0.05. Tests for a significant difference in nodular volume reduction between solid and echocomplex as well as between hot and non-hot nodules were conducted using a two-sided Wilcoxon test.

Correlations are rounded down and reported up to the second position after the decimal point; *p* values are brought up to a round figure at the second decimal place. Other values are reported up to the first decimal place rounded upwards from above 0.x5 and downwards below.

## Results

### Patients and baseline characteristics

Of the ten treated subjects, one was excluded from the current analysis as he received combined therapy with HIFU with subsequent RIT to reduce the necessary radiation dose. Data of the nine remaining patients (seven females (77.7%) with median age 52 (36–80)) who underwent HIFU treatment for one nodule each are included in this analysis. All treated nodules were benign (three hot or indifferent, six cold, four solid, five echocomplex; with pre-intervention volumes from 0.8 to 7.7 ml, median 3.5 ml). See Table 1 for more information on patients.

**Table 1:** **Patient characteristics**

**Patient number**	**Age**	**Sex**	**Clinical diagnosis**	**Previous treatment on the thyroid**	**TN-related symptoms**	**Pathological findings in FNAB**
1	50	M	Struma nodosa with hypofunctional nodule		Globus pharyngis	No indication for malignancy
2	74	F	Struma nodosa with unifocal thyroidal autonomy		Several symptoms associated with hyperthyroidism	
3	36	F	Struma nodosa with hypofunctional nodule		Swallowing disorder	No indication for malignancy
4	52	M	Struma uninodosa with unifocal thyroidal autonomy and hyperthyroidism	l-Thyroxin medication	Several symptoms associated with hyperthyroidism, globus pharyngis	
5	44	F	Thyroidea nodosa with hypofunctional nodule	l-Thyroxin medication	Swallowing disorder, discomfort, Globus pharyngis	No indication for malignancy
6	80	F	Hypofunctional nodule		Globus pharyngis	No indication for malignancy
7	62	F	Struma multinodosa with isofunctional nodule	Partial thyroidectomy 30 years ago	Sensation of throat tightness, dyspnea on exertion, swallowing disorder	
8	50	F	Hypofunctional nodule	RIT due to focal thyroidical autonomy 2 years ago	Swallowing disorder	No indication for malignancy
9	58	F	Struma nodosa with hypofunctional nodule		Globus pharyngis	No indication for malignancy

This table shows detailed information on each patient.

### Procedure characteristics

Mean treatment depth ranged from 13.6 to 24.3 mm (median 18.3 mm), treated volume per patient from 0.8 to 2.1 ml (median 1.3 ml), total energy delivery per patient from 5.7 to 12.5 kJ (median 9.9 kJ) and delivered energy per site from 113.1 to 192.8 J (median 163 J). The number of treated sites ranged from 34 to 87 (median 54). Each site was treated with a 4-s pulse; duration of inter-pulse cooling intervals was positively dependent on pulse intensity and took 20 to 40 s. A median of 45.5% of the total nodular volume could be treated (range 26.6% to 57.1%). Median treatment time was 62 min (range 42 to 96 min). Patients received therapy either under local anaesthesia with Mecain or no anaesthesia at all. Treatment was done on an outpatient basis and was completed as planned in all cases.

### Reduction of nodule volume

**Figure 3:**
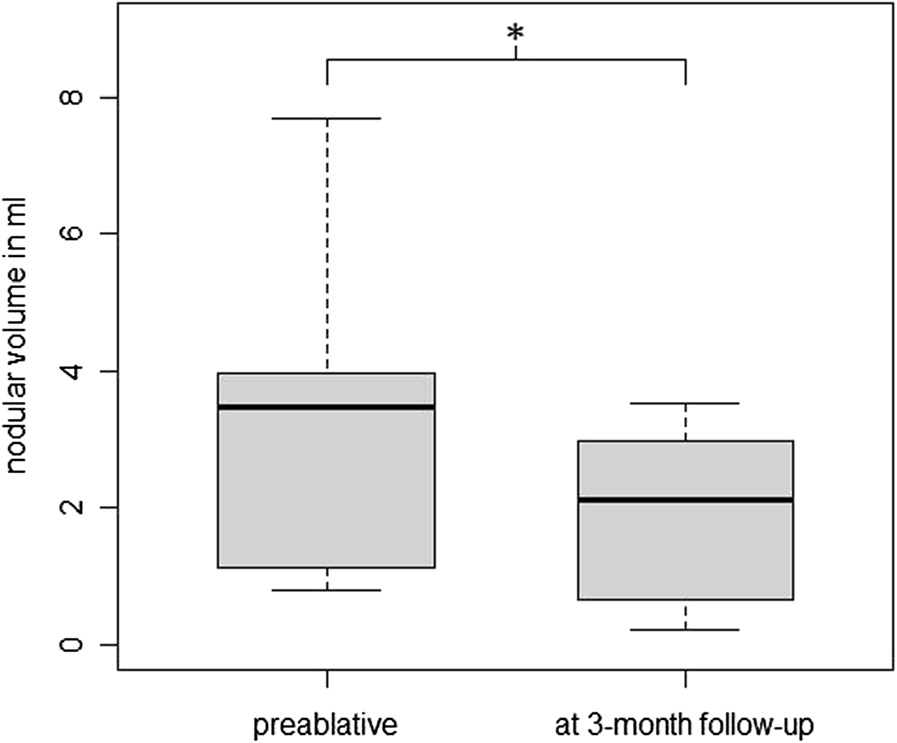
**Volume reduction.** Median nodular volume reduction within 3 months after HIFU treatment was 0.7 ml. The asterisk indicates a significant change with *p* < 0.05.

**Figure 4:**
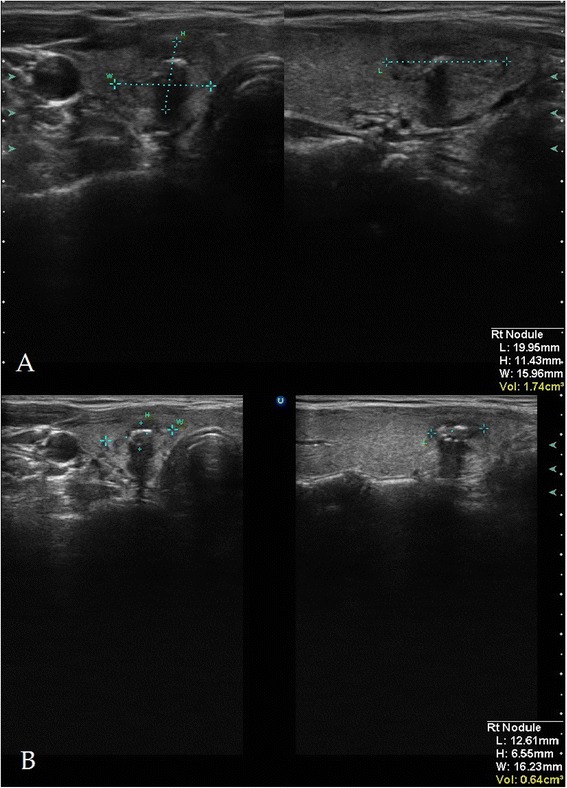
**Volume measurement.** Sonographical volume measurement of an exemplary thyroidal nodule before **(A)** and 3 months after **(B)** HIFU treatment.

All nine TNs decreased in size relative to baseline at 3 months after HIFU therapy. Absolute decrease ranged from 0.4 to 4.7 ml (median 0.7 ml, *p* = 0.01; Figures 3 and 4), and relative volume reduction ranged from 11.4% to 75% (median = 48.8%). See Table 2 for more information on the treated nodules.

**Table 2:** **Nodule characteristics**

**Patient number**	**TN location**	**Average depth of focal point during treatment in mm**	^**99m**^ **Tc-pertechnetate uptake behaviour/functionality**	**Nodular structure**	**TN volume in cm** ^**3**^		**TN height diameter in mm**		**TN width diameter in mm**		**TN depth diameter in mm**	
					**Before treatment**	**3 months after treatment**	**Before treatment**	**3 months after treatment**	**Before treatment**	**3 months after treatment**	**Before treatment**	**3 months after treatment**
1	Cranial left	24.3	Cold	Solid	1.1	0.7	12.2	13.9	13.8	10.9	13.9	10.1
2	Caudal left	18.3	Hot	Solid	0.8	0.2	13.1	5.4	9.3	5.1	13.8	12.6
3	Isthmal	13.6	Cold	Echocomplex	4.3	2.2	16.2	11.0	23.2	19.9	24.2	21.4
4	Caudal right	14.7	Hot	Echocomplex	1.7	0.6	11.4	6.6	16.0	16.2	20.0	12.6
5	Centro-cranial right	14.4	Cold	Solid	3.5	2.2	13.8	8.5	20.3	21.3	25.9	25.0
6	Cranial left	17.1	Cold	Solid	1.0	0.3	9.0	6.6	16.3	8.3	15.4	11.3
7	Centro-caudal right	21.4	Indifferent	Echocomplex	3.8	3.3	15.3	18.1	18.2	14.5	29.2	25.4
8	Caudal left	23.7	Cold	Echocomplex	7.7	3.0	21.2	8.8	25.4	27.6	29.8	25.6
9	Centro-caudal left	23.6	Cold	Echocomplex	4.0	3.5	18.5	17.2	27.8	28.4	16.1	15.0

This table shows detailed information on each treated nodule.

Amongst tested variables, correlation to absolute nodular volume reduction was significant only for mean treatment depth (*τ* = −0.61, *p* = 0.03) and scintigraphic nodular uptake reduction (*τ* = 0.66, *p* = 0.02). Uptake reduction correlated to relative nodular volume reduction with (*τ* = 0.44, *p* = 0.12).

Correlations of absolute nodular volume reduction to pre-intervention nodular volume (*τ* =0.38, *p* = 0.19), treated volume (*τ* = −0.11, *p* = 0.77), total energy delivered per nodule (*τ* = 0.05, *p* = 0.92), nodular structure (solid or echocomplex; *p* = 0.72) and nodular functionality (hot or not; *p* = 0.56) had no significant impact on volume reduction.

In three of nine TNs, volume reduction as reported in the treatment report was larger than the treated volume, and in six cases, the reverse occurred. Median treatment depth for the former was 14.4 mm and for the latter 19.9 mm.

Euthyrosis (normal ranges for TT3, TT4 and TSH are 1.0–3.3 nmol/l, 55–170 nmol/l and 0.3–4.0 mE/l, respectively) was achieved or preserved in eight cases; one patient with an autonomous hyperfunction presented a hidden hyperthyroidism as expected, as HIFU treatment was done on an additional cold TN.

### Safety

No major complications such as vocal cord palsy; tracheal, vagal or oesophageal injuries; infections; or nodule rupture were observed. Patients were asked to report the pain they felt during the interventional HIFU pulses and directly after treatment completion using a numeric rating scale reaching from 0 to 10. Median pain level during treatment beams was 5.5 (range 3 to 7), while it was only 2 (range 0 to 4) right after the completion of interventions. All patients reported discomfort on the throat due to probe pressure and cooling; most described a spreading of pain towards the neck, scapula, trapezius muscle or arm over time.

Most patients showed a slight reddening of the skin and mild swelling above the treated nodule 24 h after the treatment; one patient developed a small hematoma at the puncture location of the local anaesthesia. No patient reported any pain 24 h after treatment.

All minor complications resolved within days without needing any treatment. During the next 3 months, no further complications were observed. Informal reports indicate that acceptance of treatment amongst patients was very good.

## Discussion

### Strengths and weaknesses of HIFU

HIFU’s main advantage relative to local thermal ablation techniques like RFA or MWA is non-invasiveness. Consequently, HIFU requires less anaesthetic, makes infection highly unlikely and precludes scar formation. As such, patient acceptance of it is very high.

Non-invasiveness is also the source of HIFU’s relative weakness in that its energy must be transported through overlying, non-target tissue before reaching the ablation area, as opposed to RFA and MWA where ablation energy is applied directly to affected areas. As a result, HIFU’s main technical challenge is avoidance of damage to pre-focal tissues while delivering sufficient heating to the target volume.

HIFU’s main shortcomings, which were observed during treatment, are related to this trade-off as well, namely, the procedure’s long duration and its incomplete ablation. The amount of volume that could actually be treated in relation to initial nodular volume was limited by four built-in constrains of the EchoPulse®: (1) application to only one layer of treatment sites, (2) maximum treatment depth of approximately 2.8 cm, (3) security margins around sensible structures and (4) only one direction of site alignment of 90° to the skin.

Current experience with the device and TN treatment in general argues against changing (1) since it would lead to prolonging an already lengthy treatment whose median duration was 62 min. At the same time, removing this limitation might increase treatment coverage and, with it, considerably increase therapy effectiveness. This might be especially useful for larger TNs, since these are more likely to have relevant masses above and/or below the treatment layer.

Treatment depth limitation of HIFU has already been reported in other indications [24]. This is due to the fact that a sufficient heating of deeper layers of tissue requires larger amounts of energy to be emitted, as more of it is lost in pre-focal areas, which in turn can only handle limited heating per time unit without being damaged. The EchoPulse® addresses this problem by increasing heat flux from the non-targeted tissue the US pulse passes through by cooling the overlying skin. Yet, median treatment time is still 62 min and median ablated volume only 1.3 ml. Moreover, 3-month volume reduction of deeper lying nodules is lower relative to that of shallower nodules, with correlation of treatment depth and volume reduction being *τ* = −0.61 (*p* < 0.05). A potential solution for this could be utilization of either a larger probe or multiple probes targeting the same focal point from different angles, thus allocating energy lost in pre-focal tissue to more volume. Additionally, more flexible probe positioning may make it possible to send each pulse through a different part of overlaying tissue and hence minimize inter-pulse treatment pauses.

Yonetsuji et al. [25] suggest that an increase in degrees of probe rotation helps reduce skin burns, long treatment time and incomplete ablation, which are the main problems of HIFU therapy in general. This indicates that (4) indeed has been limiting treatment success.

Sufficient security margins (3) are an essential requirement considering our data showing effects of HIFU beyond the targeted area, as implied by nodular shrinkages larger than the treated volumes. Specifically, this was observed in three of our nine cases with reductions of 2.1, 1.4 and 4.7 ml induced when treating volumes of 1.3, 1.0 and 1.5 ml, respectively.

These off-focus lesions should be majorly situated above the focal point, as there the pulse is more powerful than below it. Parafocal lesions should occur due to heat conduction as well as convection by blood flow but should only be marginal.

The main reason for long treatment durations is that after each pulse emission, the non-targeted area around and especially above the nodule needs to cool down, which takes time increasing with the energy absorbed in pre-focal tissue. As noted, this could be addressed by larger, multiple, or more flexible probes.

### Volume reduction

Turtulici et al. [12] found that a single RFA treatment of larger benign non-functioning TNs (mean pre-ablation volume 13.5 ml) could induce mean relative shrinkage of 72.6% after 6 months. Baek et al. [26] observed average decreases of 82.6% for predominantly solid benign TNs after the same period.

Ha et al. [27] who used HIFU for nodules with pre-ablative volume of 9.7 ml were able to induce a mean reduction of 87.2% after an average of 43.7 months. A meta-analysis by Shin et al. [22] demonstrated that a single RFA session induces reduction of 33% to 49% in predominantly solid TNs after 1 month and 51% to 80% after 6 months.

Korkusuz et al. [7] report volume reductions of 54.2% 3 months after MWA in benign cold TNs. Yue et al. [28] induced shrinkages of 65% on average using MWA, measured 6 months after ablation.

Døssing et al. [10] report a median reduction of the solid component volume of a cystic TN of 54% 6 months after a combined therapy of cyst aspiration and subsequent ILP. They also produced a median volume reduction of benign cold solid TNs of 51% a median of 67 months after a single ILP session [29]. They also compare ILP and RIT on solitary autonomous nodules and found similar volume reductions after 6 months on average of 44% and 47%, respectively [30].

Compared to these values, the findings of this study regarding a relative median shrinkage of 48.8% of relatively small TNs after only 3 months are decent, especially considering the technical constraints explained above that were limiting treatment coverage.

### Factors influencing success

As deeper lying TNs presented significantly smaller volume reductions (*τ* = −0.61, *p* < 0.05), pre-focal energy losses appear to have been higher than anticipated. Continuous intra-procedure power adjustment by “heat bubble” observation, as opposed to baseline only, might enable sufficient focal heating by real-time treatment effect visualization.

The unexpected lack of relationship between planned treatment volume and volume reduction may have been due to patient movement causing site blurring (e.g. swallowing can create tissue movement in a cranio-caudal direction that does not necessarily induce laser-detectable skin movement above 1 mm in a lateral or dorso-frontal direction). Site blurring may cause energy pulses to be distributed across a larger area than planned as opposed to being focused at a single point. This then results in lack of sufficient heating of the targeted area, leading to suboptimal ablation.

The six cases in which nodular shrinkage was lower than the treated volume do not necessarily need to be explained by effectiveness-reducing effects like site blurring or lower effectiveness in deeper lying TNs, as post-ablative nodular shrinkage is not usually completed after 3 months.

The correlation of baseline nodular volume (*τ* = 0.38, *p* > 0.05) with nodular shrinkage might be due to two different effects: off-focus lesions are more likely to lie within the target TN if it is larger, and larger TNs obviously have more potential for shrinkage measured in absolute volume reduction. The reason for the correlation being neither high nor significant could be explained by interfering effects such as site blurring, strong relevance of treatment depth and small sample size.

The near non-correlation of total energy delivered per nodule to nodular volume reduction (*τ* = 0.05, *p* > 0.05) can be explained by this as well.

Cystic nodule shrinkage is generally better. Sung et al. [31] report mean reduction values for such TNs at the 6-month follow-up of 96.9% and 93.3% after a single session of EA or RFA, respectively.

In this study, however, partially cystic “echocomplex” nodules did not yield significantly different volume reductions compared to solid ones (*p* > 0.05). In RFA and MWA, cysts within, or even only infringing the effect radius, should be sufficiently heated to ablate the adjacent cells. With HIFU, however, only small doses of energy are emitted per time. If, via heat conduction from neighbouring tissue, this energy is distributed within a larger volume of cyst liquid, threshold temperatures may never be reached.

Possibly, most cysts within “echocomplex” TNs were too small for such effects to take place, as singular pulses were able to deliver sufficient energy per micro-cyst. In this study, all echocomplex TN presented a cystic component of less than 25% of the total nodular volume.

Functional activity of thyroid tissue is independent of its sensitivity to heating. As expected, hot nodules did not yield significantly different volume reduction than cold or indifferent ones (*p* > 0.05).

### Treatment success prediction

Previous studies have shown that neither US elastography [32,33] nor colour-coded duplex US [34] can replace scintigraphy for assessing TNs, especially hot ones. The relative uptake reduction values of this study were already reported earlier [35] and ranged from 10% to 57%. This is proof of normal thyroidal tissue lying in front and/or behind the treated nodule remaining functionally intact.

The findings of this study suggest that the relative target area background adjusted uptake reduction in relation to total thyroidal uptake predicts nodular shrinkage to a certain extent (*τ* = 0.66, *p* < 0.05). This is plausible as uptake reductions are due to induced necrosis, which in turn will be disintegrated over time and leads to nodular volume reduction. The reason for uptake reduction correlating with absolute rather than relative volume reduction (*τ* = 0.44, *p* > 0.05) may be due to two reasons. Firstly, relative uptake reductions themselves are not linearly dependent on successful treatment coverage, as scintigraphic imaging is two-dimensional, and thus, uptake in tissue lying in front or behind the nodule is nevertheless registered as lying in the target area. Secondly, Kendall’s *τ* is a non-parametric rank correlation coefficient, and ranks between absolute and relative volume reduction may change. In small samples, a single such change might cost significance.

## Conclusion

HIFU treatment of benign predominantly solid TNs is easy to perform, non-invasive and appears to be safe. There is, nevertheless, room for its improvement in terms of technical optimization to further increase treatment coverage and decrease duration. With the current technology, the method is already able to produce relative nodular volume reduction rates similar to more established techniques for local ablation. Considering HIFU’s advantages of non-invasiveness, lower infection risk and no scar formation, the method should be further explored. Especially for the treatment of small shallow benign TNs, HIFU might prove to be superior to alternative treatments. Specific issues that should be addressed in the future include exploration of the possible benefits of multiple procedures and long-term outcome.

It is nevertheless important to note that this study’s finding and conclusions are based on a small sample and should be validated in larger trials.

## Abbreviations

HIFU: High-intensity focused ultrasound
TN: Thyroidal nodule
US: Ultrasound
FNAB: Fine-needle aspiration biopsy
RIT: Radioiodine therapy
ILP: Interstitial laser photocoagulation
RFA: Radio-frequency ablation
MWA: Microwave ablation
EA: Ethanol ablation
